# Targeting beta-lactamase activity with Oxacyclohexadecan-2-one in carbapenem-resistant uropathogenic *E*. *coli*: A molecular simulation approach

**DOI:** 10.1371/journal.pone.0317941

**Published:** 2025-02-18

**Authors:** Priyanka Balaji, Madhana Priya N, Emmanuel Bhaskar M., Gnanasambandan R, Solomon F. D. Paul, Magesh R.

**Affiliations:** 1 Department of Human Genetics, Faculty of Biomedical Sciences and Technology, Sri Ramachandra Institute of Higher Education and Research, Chennai, Tamil Nadu, India; 2 Department of Biotechnology, Faculty of Biomedical Sciences and Technology, Sri Ramachandra Institute of Higher Education and Research, Chennai, Tamil Nadu, India; 3 Department of Biotechnology, Faculty of Science and Humanities, SRM Institute of Science and Technology- Ramapuram, Chennai, Tamil Nadu, India; 4 Department of General Medicine, Sri Ramachandra Institute of Higher Education and Research, Chennai, Tamil Nadu, India; 5 Department of Biomedical Sciences, School of Biosciences and Technology, Vellore Institute of Technology, Vellore, Tamil Nadu, India; Regional Medical Research Centre Bhubaneswar, INDIA

## Abstract

Urinary tract infections caused by uropathogenic *Escherichia coli* (*E*. *coli)* are a global health concern, with rising rates and antibiotic resistance demanding novel treatments. Therefore, in this study, we explored the potential of Oxacyclohexadecan-2-one obtained from *Moringa oleifera (M*. *oleifera)* seed, as antibacterial agent against three majorly prevalent carbapenemase-producing *E*. *coli* proteins, blaNDM-1 (New Delhi metallo-betalactamase-1), blaNDM-5 (New Delhi metallo-betalactamase-5) and blaOXA-48 (Oxacillinase-48) from the strains Ecw3, EC-114 and T20 respectively. The ethanolic extract of *M*. *oleifera* seed was subjected to GC-MS, identifying 135 compounds. PyRx virtual screening, identified the top 10 ligands for each protein following the Rule of 5 and ProTox classes V and VI, with Oxacyclohexadecan-2-one (PubChem ID: 235414) showing best binding affinity across all 3 proteins with an optimized dose (LD50) of 5000mg/kg. Hence, molecular docking was carried out for ligand 235414 along with Imipenem, belonging to the same class V toxicity class with an optimized dose (LD50) of 5000mg/kg. Imipenem is a commonly used FDA drug to treat UTIs, which served as the control in the study. Oxacyclohexadecan-2-one showed higher binding affinity for the beta-lactamase proteins with a docking score of -6.45 kcal/mol, -6.05 kcal/mol and -7.34 kcal/mol compared to -3.41 kcal/mol, -3.99 kcal/mol and -6.36 kcal/mol of Imipenem for NDM-1, NDM-5 and OXA-48 respectively. Dynamic Simulation was performed for 100 ns for Oxacyclohexadecan-2-one and Imipenem bound protein complexes to determine the stability, fluctuations, compactness, bond interaction, solvent accessibility area, free energy landscape and the binding free energy. The results of molecular docking and dynamics were promising for the Oxacyclohexadecan-2-one, suggesting its potent inhibitory effect against the beta-lactamase producing proteins.

## Introduction

Urinary Tract Infections (UTIs) are prevalent globally, affecting almost 150 million people every year with substantial morbidity and high medical cost [1]. Severe complications, including septic shocks, acute kidney injury and pyelonephritis, result in around 230,000 deaths annually [2]. Various factors contribute to UTIs, notably age and comorbidities like diabetes mellitus [3]. In regions like India, with a high prevalence of diabetes, incidence of UTIs is expected to increase. Uropathogenic *Escherichia coli* (UPEC) is the most common pathogen, causing approximately 50–75% of UTIs, followed by *Klebsiella pneumoniae* at 10–15% [4]. With rising UTI rates globally and increasing antibiotic resistance, novel pharmacological treatment strategies are imperative, particularly in diabetic populations, where antibiotic resistance is heightened. Chronic high blood glucose levels, impair mucosal barriers making individuals more susceptible to UTIs and recurrent UTIs (rUTIs). Adding to this challenge, most pathogens causing UTIs in diabetic population are multi-drug resistant (MDR). A cross-sectional study conducted in Ethiopia involving diabetic patients with symptomatic UTI reported a MDR rate of 81.1% [5]. MDR uropathogens are resistant to at least one drug in 3 or more antimicrobial categories.

As UTIs are currently only being treated with classes of antibiotics such as Ampicillin, Nitrofurantoin and Carbapenems, continuous usage of these can lead to development of resistance as the pathogens adapt to the toxicity of modern medicines. The common uropathogen *E*. *coli*, is well-known for its capacity to acquire genes that aid in antibiotic resistance. And uropathogenic *E*. *coli* isolated from UTI-infected diabetic individuals, show resistance to most studied antibiotics, such as co-trimoxazole, nitrofurantoin, ampicillin, and ciprofloxacin. The most reported proteins expressed by *E*. *coli* that confer resistance to a broad-range of carbapenem drugs are NDM, OXA, TEM, CTX-M, VIM, and IMP [6]. These proteins produce an enzyme, beta-lactamase that neutralizes the beta-lactam ring of the carbapenem drugs, thus rendering the drug inactive [7].

To address UTI challenges and to reduce the complete burden on antibiotics for the treatment of UTIs, plant-derived compounds such as proanthocyanidins, probiotics, phenolic compounds, flavonoids, alkaloids, and D-Mannose are being studied [8]. Plant extracts could provide a valuable alternative to synthetic drugs in treating UTIs, as they contain a variety of bioactive compounds that target bacteria through multiple mechanisms, thereby reducing the likelihood of resistance development [9–12]. Plant-based compounds also have fewer side effects, offering a sustainable and safer option for prolonged treatment, especially in populations with recurrent infections.

*Moringa oleifera (M*. *oleifera)*, a plant native to the Indian subcontinent, possesses antioxidant and anti-inflammatory properties, with studies suggesting its efficacy in managing diabetes and infections [13–15]. The bark extracts of *M*. *oleifera* have exhibited a cure rate of 66% compared to standard antibiotics in treating UTIs [16]. Apolar fraction of *M*. *oleifera* seed also has proven to exhibit antibacterial activity against certain gram-negative bacteria such as *Staphylococcus aureus* and *Staphylococcus epidermidis* [17].

Computational aided drug discovery (CADD) uses AI-driven modeling and data analysis to optimize drug candidates, crucial in combating antibiotic resistance [18, 19]. These technologies also offer effective virtual-screening of large chemical libraries to identify novel possible antibacterial chemicals. Ideal drug candidates with high binding affinity and specificity for bacterial pathogens can be identified by computationally analyzing the interactions between small molecules and biological targets considering the molecular structure, electrostatic characteristics and binding energies. This enables antibacterial activity to be predicted prior experimental validation. Therefore, our study aims to screen *M*. *oleifera* seed-derived compounds against the commonly reported carbapenem-resistant proteins NDM-1, NDM-5, and OXA-48 of uropathogenic *E*. *coli*. By identifying potential antibacterial agents from natural sources, we hope to contribute to the development of novel therapeutic strategies to combat these disease-causing pathogens.

## Methodology

### Preparation of target protein molecules

The three-dimensional crystallographic protein structures of the most reported carbapenem-resistant proteins NDM-1, NDM-5 and OXA-48 of *E*. *coli* across all strains were extracted from the RCSB Protein Data Bank (https://www.rcsb.org/). Further, using the PyMol Molecular Graphics System version 3.0.0 (https://www.pymol.org/) software. All the non-interacting ions and water molecules of the proteins were removed using PyMol and the structure of the protein was saved in PDB format [20, 21].

### Identification of the binding site

An online tool, Computed Atlas of Protein Surface Topography (CASTp) was used to determine the surface properties and functional areas of the proteins [22]. The PDB file of the native protein was uploaded and submitted (http://sts.bioe.uic.edu/castp/index.html?j_6639d9c49ab68). Within the active site of the protein, the residues were displayed along with the surface area and volume. For rigid docking, the binding site residues corresponding to the ligand were chosen.

### Preparation of ligand molecules

The *M*. *oleifera* seeds were dried, ground, extracted and screened through GC-MS for the retrieval of the phyto-components. The listed compounds were identified and the SDF files of their 3-dimensional structures were obtained from the PubChem database (S1 Data). The OpenBabel tool converted all the ligands to PDBQT format [23].

### Virtual screening of compounds using PyRx

The crystal-structure of the protein was prepared by adding Kollman charges, hydrogen atoms and gasteiger using Auto Dock Tools (ADT). Only the polar hydrogen bonds remained distributed over the protein. These dockable proteins were stored as.pdbqt files for further screening. Energy minimization of the compounds was performed and converted to PDBQT files. The energy values were analyzed through the Auto Dock Vina wizard option of the PyRx software. PyRx is a virtual drug-screening software which is used to screen compound libraries against respective drug targets [24]. Exhaustiveness of 8 was maintained to screen all the GC-MS derived compounds from PubChem database and subsequently, the top 10 compounds were considered for further analysis.

### Pharmacokinetic analysis

SwissADME, an online software tool was used to estimate variables such as the absorption, distribution, metabolism, and elimination (ADME) of physicochemical features and to determine the recommended daily dosage and bioavailability of the compound (http://www.swissadme.ch/index.php). Using the tool, only the ligands which follow the Lipinski rule are further considered for docking studies, ADME properties of the compounds was identified with the canonical SMILES of each compound as input obtained from PubChem. The list displaying criteria such as Lipinski rule, molecular weight, H-bond acceptors and donors, rotatable bonds and logP value etc are shown and noted.

### Toxicity analysis by ProTox

ProTox is an online toxicity assessment tool that shows the toxicity level of a compound. An input of a compound is given either by the compound’s canonical SMILES form or the PubChem name. When the toxicity prediction is performed, the results are displayed in the form of toxicity class, and compounds which fall under the class 5 and 6 are only considered for further docking analysis [25].

### Docking studies

Molecular Docking was performed to determine the best fit between two molecules, which resembles a lock and key mechanism. Based on the binding affinity of the target protein with the ligand, this method projects the 3D structure of a complex. The software used for performing docking is the Auto Dock tool, version 1.5.7 [26]. First, Auto Grid is performed by choosing the protein and the map-types are set by selecting the ligand molecule. The corresponding binding site residues are selected for the protein molecule. Successful completion of Auto Grid indicates that the molecule is ready for docking. For docking, the protein is set as rigid filename and ligand molecules are chosen. After checking the genetic algorithm and docking parameters, Auto Dock is run. The docking files are saved as.dlg. To ensure a representative examination, docking is performed in triplicates. The interactions of the protein-ligand are then visualized using the BioVia Discovery Studio tool.

### Molecular Dynamics Simulation (MDS)

MDS was performed for the protein-IPM and protein-test ligand complexes using the GROMACS 4.5.4 suite to better understand the ligand’s interaction with the protein’s functional area [27]. Protein and ligand topology files were created and fixed in the center of a dodecahedron box with water molecules. The system was compensated by the addition of counter-ions. Utilizing the steep descent algorithm and conjugate gradient methods, the energy of the complex system was decreased. To achieve a stable condition, the NVT and NPT were counterbalanced for the overall system energy. After selecting the parameters for the temperature as 300K and pressure as 1 bar, the 100 ns MD simulation was initiated, followed by Root Mean Square Deviation (RMSD), Root Mean Square Fluctuation (RMSF), Radius of Gyration (Rg), Hydrogen Bond (H-bond), MM/PBSA, and SASA analysis of the MD trajectory files.

To determine the protein-ligand complex stability in motion, Principal component analysis (PCA) was also performed. PCA identifies the key determinants of a protein’s trajectory based on changes and covariance on the protein backbone. To generate the variance and covariance backgrounds gmx covar module was used and gmx anaeig module, to identify changes and significant movements of protein structures. The input files were given as eigenvectors and eigenvalues, and the RMSF/atom eigenvectors were shown as output. The first and second projections of the protein trajectory were depicted as a graph against a dynamic run of 100 ns. To determine the binding energy of beta-lactamase protein and ligand complexes, molecular mechanics/Poisson-Boltzmann surface area (MM/PBSA) were carried out. The MM-PBSA computations represent the summary of the binding free energy of biomolecular interactions between beta-lactamase proteins and ligands in the last 50 ns [28, 29].

## Results

### Target protein data collection and identification of the binding site

**Fig 1:**
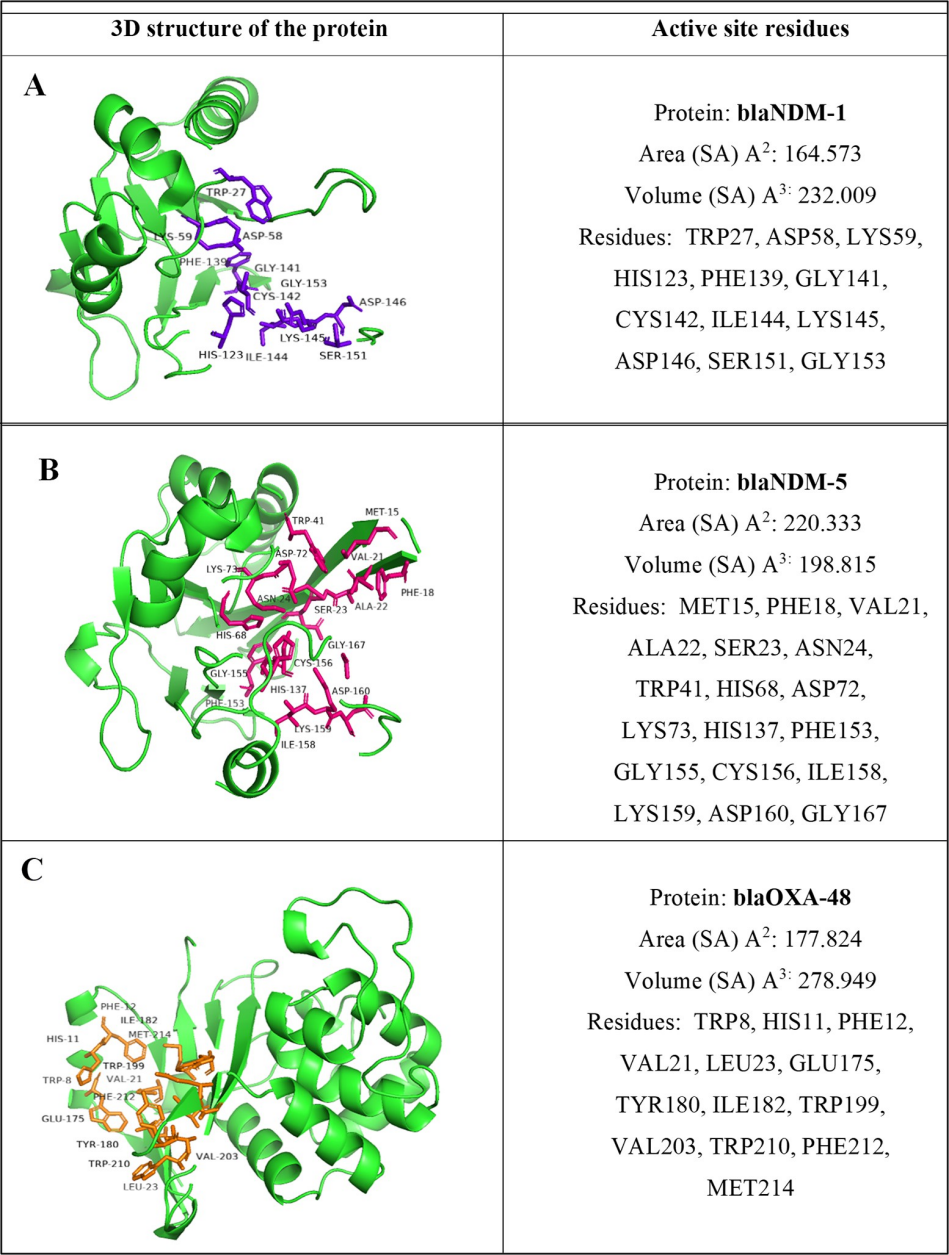
(A-C). The 3-dimensional structure of the antimicrobial resistant target proteins. The active site residues are displayed with the surface area and volume.

The 3D protein structures of blaNDM-1, blaNDM-5 and blaOXA-48 with IDs G3K399_ECOLX, A0A290DNG8_ECOLX and A0A0M5JXH3_ECOLX respectively were extracted from UniProt and modeled using the SwissModel tool. CastP identified the active site region of the NDM-1, NDM-5 and OXA-48 proteins as, 164.573SA, with volume 232.009SA, 220.333SA with a volume of 198.815 and 177.824SA with a volume of 278.949SA respectively (Fig 1A–1C).

### Virtual-screening of the retrieved ligand molecules

The 135 ligand molecules obtained from the PubChem database were virtually-screened with each of the protein. The top 10 hits with best binding affinity (low energy values in kcal/mol and zero RMSD) were selected. The ligands with respective binding affinity with each of the proteins were tabulated (Tables 1–3).

**Table 1:** Top 10 hits of *M*. *oleifera* seed compounds identified after virtual screening with blaNDM-1 protein along with their ADME(T) properties.

PubChem ID	Name of the compound	Binding affinity (kcal/mol)	Lipinski Rule of Five	ProTox Class
70354	Oxacyclotridecan-2-one	-5.6	PASS	V
15596	Oxacyclotetradecan-2-one	-5.5	PASS	V
**235414**	**Oxacyclohexadecan-2-one**	**-5.4**	**PASS**	**V**
531620	4-Cyanobenzoic acid, undec-10-enyl ester	-5.3	PASS	VI
5353115	4-cyanobenzoic acid, undec-2-enyl ester	-5.1	PASS	V
5367782	2,6-Dodecadien-1-ol, 3,7,11-trimethyl-, (E, E)-	-5.1	PASS	V
5367860	(Z)-9-Hydroxy-2,4-dimethyl-non-7-enoic acid lactone	-4.9	PASS	VI
582477	Formamide, N-(4-[2-(1,1-dimethylethyl)-5-oxo-1,3-dioxolan-4-yl] butyl)	-4.9	PASS	IV
609757	4-Nitro-5-hydroxy-1,2-dimethylindole	-4.7	PASS	IV
530600	4-Cyanobenzoic acid, 4-hexadecyl ester	-4.3	FAIL	-

**Table 2:** Top 10 hits of *M*. *oleifera* seed compounds identified after virtual screening with blaNDM-5 protein along with their ADME(T) properties.

PubChem ID	Name of the compound	Binding affinity (kcal/mol)	Lipinski Rule of Five	ProTox Class
565086	3-Furan-2-yl-5-(1-hydroxy-cyclohexyl)-isoxazolidin-5-ol	-6.5	PASS	IV
550069	d-Mannitol, 1,1’-O-1,16-hexadecanediylbis-	-5.8	FAIL	-
566676	2H-Cyclohepta[b]furan-2,8-dione, octahydro-, (3aalpha,8aalpha)-	-5.7	PASS	IV
530600	4-Cyanobenzoic acid, 4-hexadecyl ester	-5.6	FAIL	-
5353115	4-cyanobenzoic acid, undec-2-enyl ester	-5.6	PASS	V
609757	4-Nitro-5-hydroxy-1,2-dimethylindole	-5.6	PASS	IV
6422771	l-Alanine, N-propoxycarbonyl-, isohexyl ester	-5.6	PASS	IV
**235414**	**Oxacyclohexadecan-2-one**	**-5.5**	**PASS**	**V**
90470748	Methyl (2R,4R,6R,8R)-2,4,6,8tetramethyloctacosanoate	-5.5	FAIL	-
15596	Oxacyclotetradecan-2-one	-5.4	PASS	V

**Table 3:** Top 10 hits of *M*. *oleifera* seed compounds identified after virtual screening with blaOXA-48 protein along with their ADME(T) properties.

PubChem ID	Name of the compound	Binding affinity (kcal/mol)	Lipinski Rule of Five	ProTox Class
**235414**	**Oxacyclohexadecan-2-one**	**-6.9**	**PASS**	**V**
609757	4-Nitro-5-hydroxy-1,2-dimethylindole	-6.4	PASS	IV
5367782	2,6-Dodecadien-1-ol, 3,7,11-trimethyl-, (E,E)-	-6.2	PASS	V
15596	Oxacyclotetradecan-2-one	-6.1	PASS	V
565086	3-Furan-2-yl-5-(1-hydroxy-cyclohexyl)-isoxazolidin-5-ol	-6.1	PASS	IV
531620	4-Cyanobenzoic acid, undec-10-enyl ester	-6.1	PASS	VI
560276	Methyl 2,6,10-trimethyltridecanoate	-6.1	FAIL	-
582153	Cyclohexanecarboxylic acid, tetradecyl ester	-6	FAIL	-
530600	4-Cyanobenzoic acid, 4-hexadecyl ester	-5.9	FAIL	-
6422783	l-Alanine, N-propoxycarbonyl-,heptadecyl ester	-5.9	FAIL	-

### ADME and toxicity analysis

The top 10 hits for each protein were further screened using the Swiss-Absorption, Distribution, Metabolism and Excretion (ADME) tool for their pharmacokinetic properties and later analyzed for their toxicity using the ProTox tool. Out of the them, one common compound was chosen as the test ligand (PubChem ID: 235414, Oxacylcohexadecan-2-one) with respect to the pharmacokinetic properties and toxicity class (Table 4). This was further used for docking studies.

**Table 4:** The SwissADME parameters of the chosen compound Oxacyclohexadecan-2-one with the optimized dose range (LD50) value-.

Compound	Molecular weight (kDa)	Hydrogen acceptors	Hydrogen donors	Rotatable bonds	LogP value	LD50
Oxacyclohexadecan-2-one	240.38	2	0	0	3.25	5000mg/kg

### Molecular docking of Oxacyclohexadecan-2-one and imipenem with beta-lactamase proteins of *E*. *coli*

Molecular docking was performed in Auto Dock prior to which H2O molecules and heteroatoms were removed from the structures. The top best compound, Oxacyclohexadecan-2-one, interacted with blaNDM-1, blaNDM-5 and blaOXA-48 at -6.45 kcal/mol, -6.05 kcal/mol and -7.34 kcal/mol respectively. Imipenem (IPM), the standard ligand, obtained docking scores of -3.41 kcal/mol, -3.99 kcal/mol and -6.36 kcal/mol for NDM-1, NDM-5 and OXA-48 respectively (Table 5). IPM is the standard beta-lactam drug, and it belongs to the carbapenem class, which has the highest antibiotic resistance in *E*. *coli* UTI cases. The two compounds, Oxacyclohexadecan-2-one, and the conventional drug IPM, were investigated further using Molecular Dynamic Simulation.

**Table 5:** Molecular docking scores obtained from Auto Dock software for the three antimicrobial resistant proteins with Imipenem and the test ligand (Oxacyclohexadecan-2-one) with their 2D interactions.

Antibiotic resistance gene	Ligand/Drug	Docking scores (kcal/mol)	Residue	Type of bond
blaNDM-1	Imipenem	-3.41	ALA138, GLY140	Hydrogen
			PHE139, ILE144	Alkyl
	Oxacylcohexadecan-2-one	-6.45	GLY11	Hydrogen
			ALA26, ILE35	Alkyl
blaNDM-5	Imipenem	-3.99	ASN24, ALA152, GLY154, GLY155	Hydrogen
			PHE153	Alkyl
			GLN8	Sulfur-x
	Oxacylcohexadecan-2-one	-6.05	ASN24, LYS73, CYS156	Hydrogen
blaOXA-48	Imipenem	-6.36	MET92, LEU173, LYS185, THR186	Hydrogen
			MET172, THR174	Alkyl
			ILE89, LYS93	Carbon-hydrogen
	Oxacylcohexadecan-2-one	-7.34	THR174, TRP199	Hydrogen
			LYS93	Alkyl

**Fig 2:**
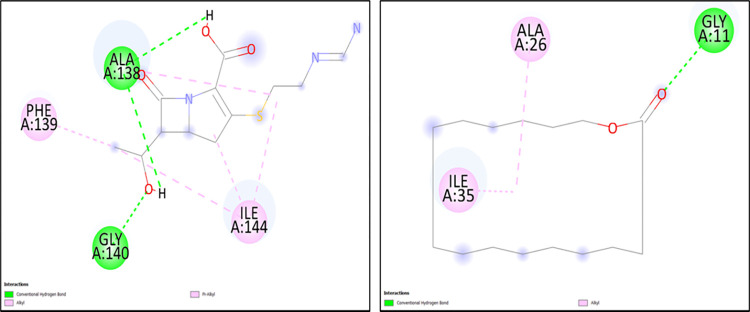
The 2D molecular docking interactions of blaNDM-1 protein with (A) Imipenem (B) Oxacyclohexadecan-2-one.

**Fig 3:**
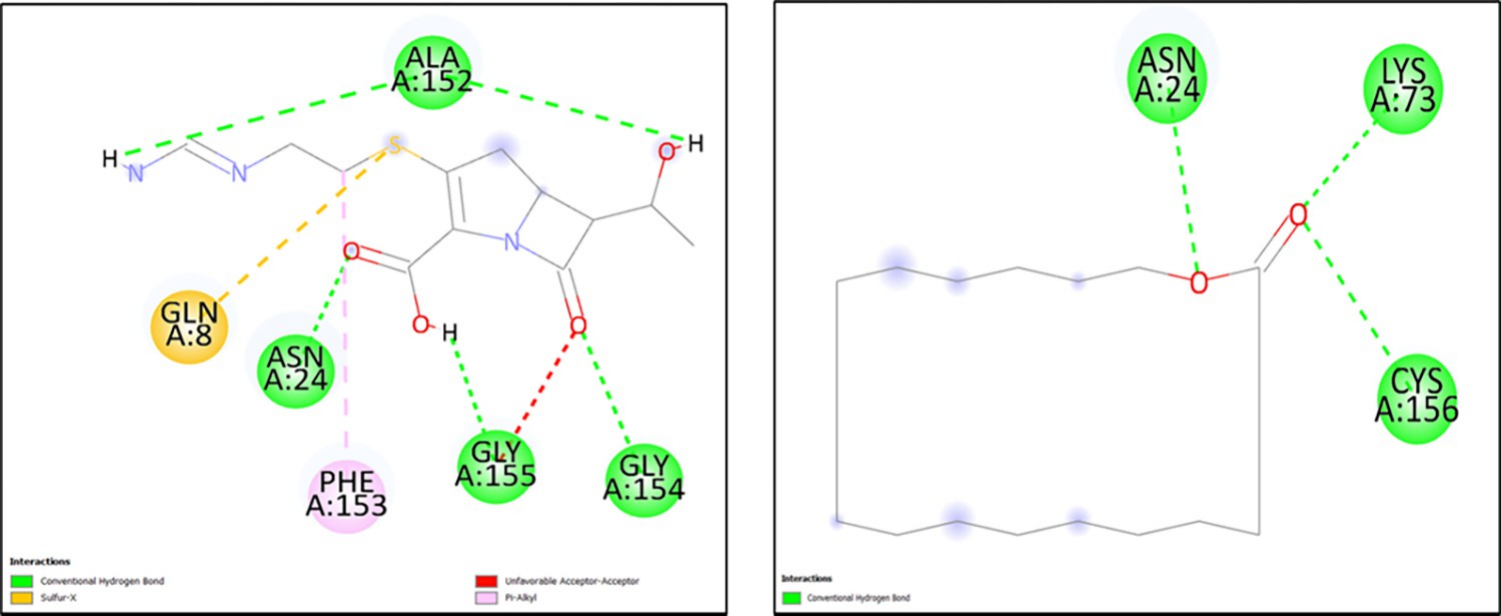
The 2D molecular docking interactions of blaNDM-5 protein with (A) Imipenem (B) Oxacyclohexadecan-2-one.

**Fig 4:**
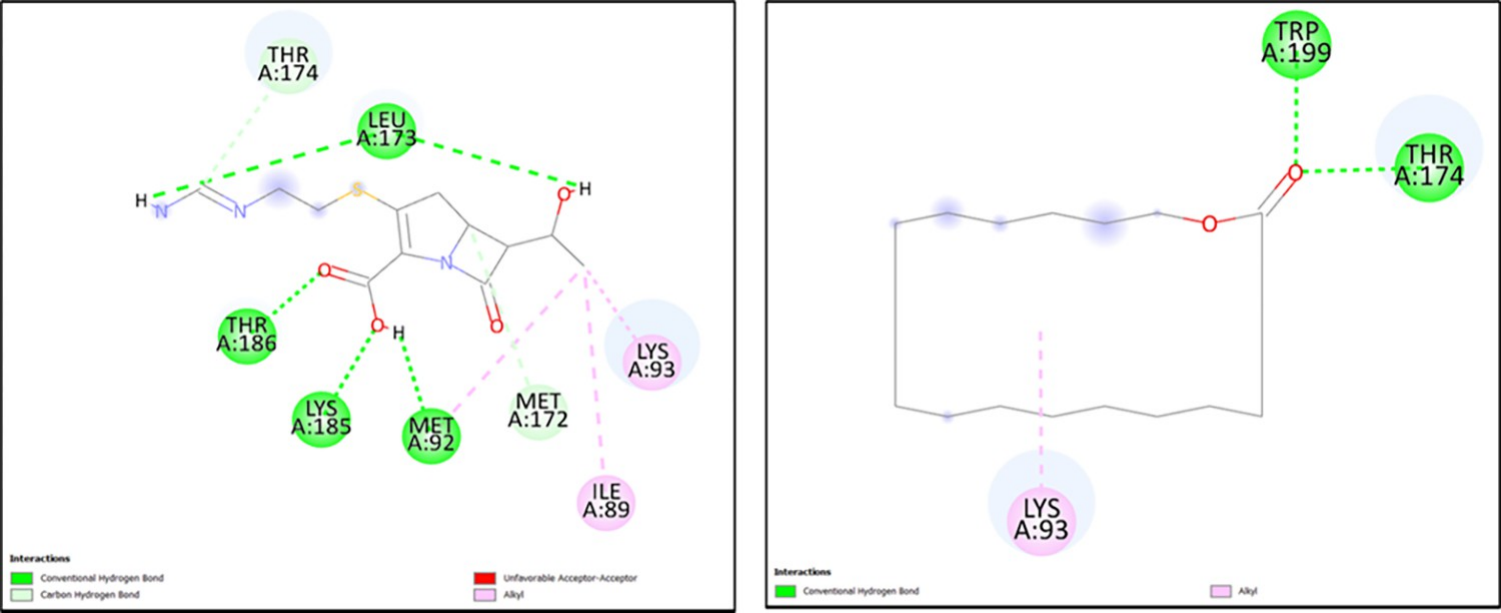
The 2D molecular docking interactions of blaOXA-48 protein with (A) Imipenem (B) Oxacyclohexadecan-2-one.

The two-dimensional figures are generated via Discovery Studio visualizer to infer the 2D ligand-interactions of molecular docking. The NDM-1 protein with Imipenem showed 2 hydrogen bonds (ALA138, GLY140), 1 alkyl (PHE139) and 1 pi-alkyl bond (ILE144), whereas with the test ligand (Oxacyclohexadecan-2-one), 1 hydrogen bond (GLY11) and 2 alkyl bonds (ALA26, ILE35) were observed. Similarly, the NDM-5 exhibited 4 hydrogen bonds (ASN24, ALA152, GLY154, GLY155), 1 sulfur-X bond (GLN8) and 1 pi-alkyl bond (PHE153) with Imipenem and 3 hydrogen bonds (ASN24, LYS73, CYS156) with the test ligand. The OXA-48 protein generated 4 hydrogen bonds (MET92, LEU173, LYS185, THR186), 2 carbon-hydrogen bonds (MET172, THR174) and 2 alkyl bonds (ILE89, LYS93) with Imipenem, likewise with the test ligand 2 hydrogen bonds (THR174, TRP199) and 1 alkyl bond (LYS93) were observed (Figs 2–4).

### Molecular Dynamics Simulation

**Fig 5:**
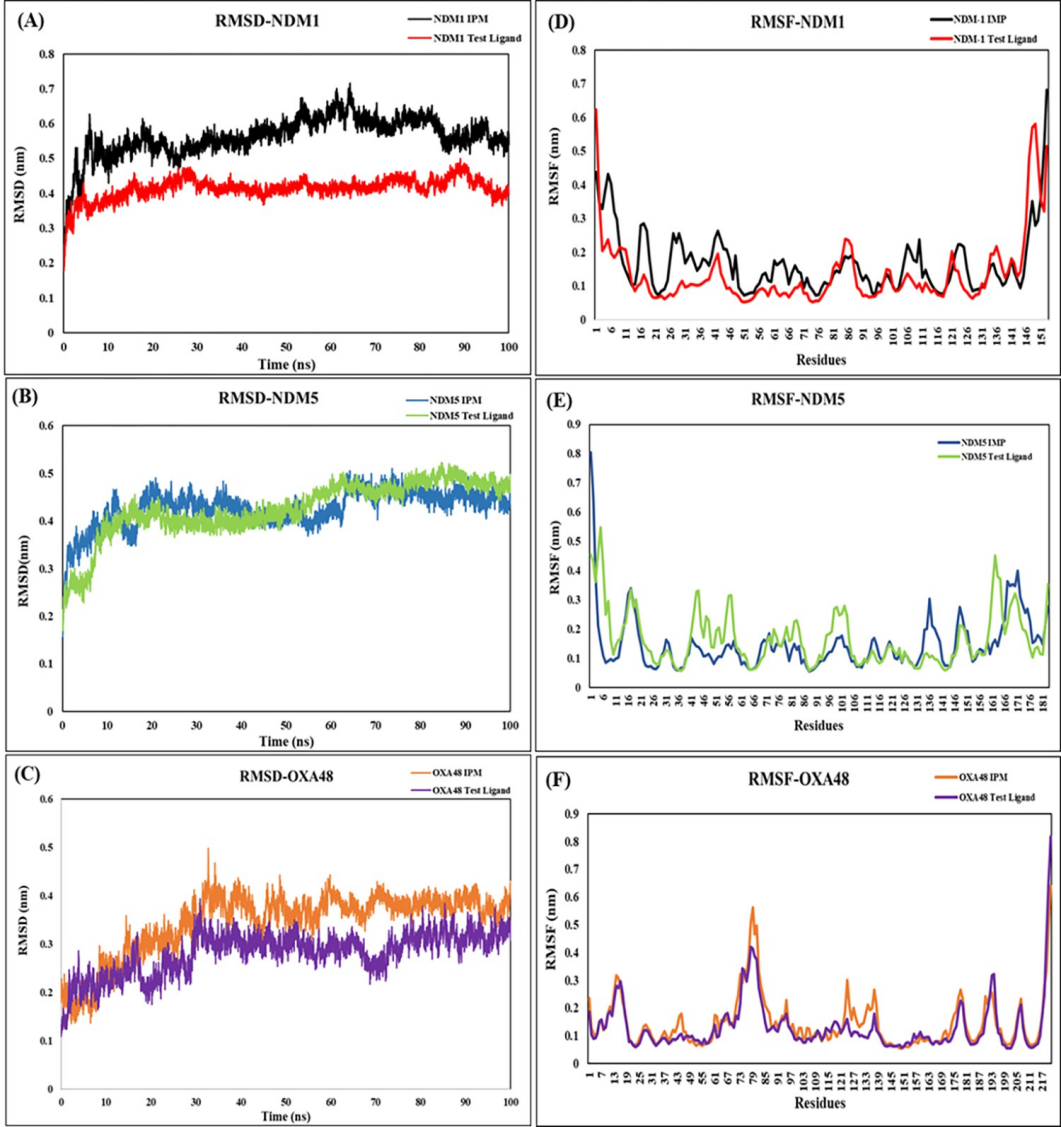
The RMSD and RMSF plots of the protein-ligand complexes for 100ns. (A) RMSD of NDM-1 (B) NDM-5 and (C) OXA-48 protein complex with IPM and test ligand. RMSF residual fluctuations of (D) NDM-1 (E) NDM-5 and (F) OXA-48 protein complex with IPM and test ligand.

MDS was run at 100 ns for all the three protein-ligand complexes. The RMSD for the six combinations of protein-ligand complexes are represented (Fig 5A–5C). The stability of the protein-ligand complex is determined by the RMSD value which explains the deviation and positional changes of the ligand molecule for each protein, when bound. The mean RMSD for the test ligand with proteins NDM-1, NDM-5 and OXA-48 was found to be 0.41 nm, 0.42 nm, and 0.28 nm respectively. Whereas, the RMSD values for IPM compared against NDM-1, NDM-5 and OXA-48 protein molecules were observed to be higher than the test ligand (Oxacyclohexadecan-2-one) with the RMSD values being recorded as 0.56 nm, 0.43 nm, and 0.34 nm respectively.

The flexibility and fluctuations of the individual residues, RMSF was calculated. The RMSF of the test ligand was observed to be 0.13 nm, 0.16 nm and 0.13 nm for NDM-1, NDM-5 and OXA-48 respectively. On the other hand, the RMSF observed for the standard drug Imipenem was found to be 0.16 nm, 0.14 nm and 0.14 nm respectively for all the three protein molecules (Fig 5D–5F). The RMSF fluctuation pattern was similar for the test ligand with NDM-1 and OXA-48 protein molecules, whereas the interaction with NDM-5 observed a higher fluctuation with the test ligand. Compared to the standard drug, the fluctuations of the test ligand were significantly low.

**Fig 6:**
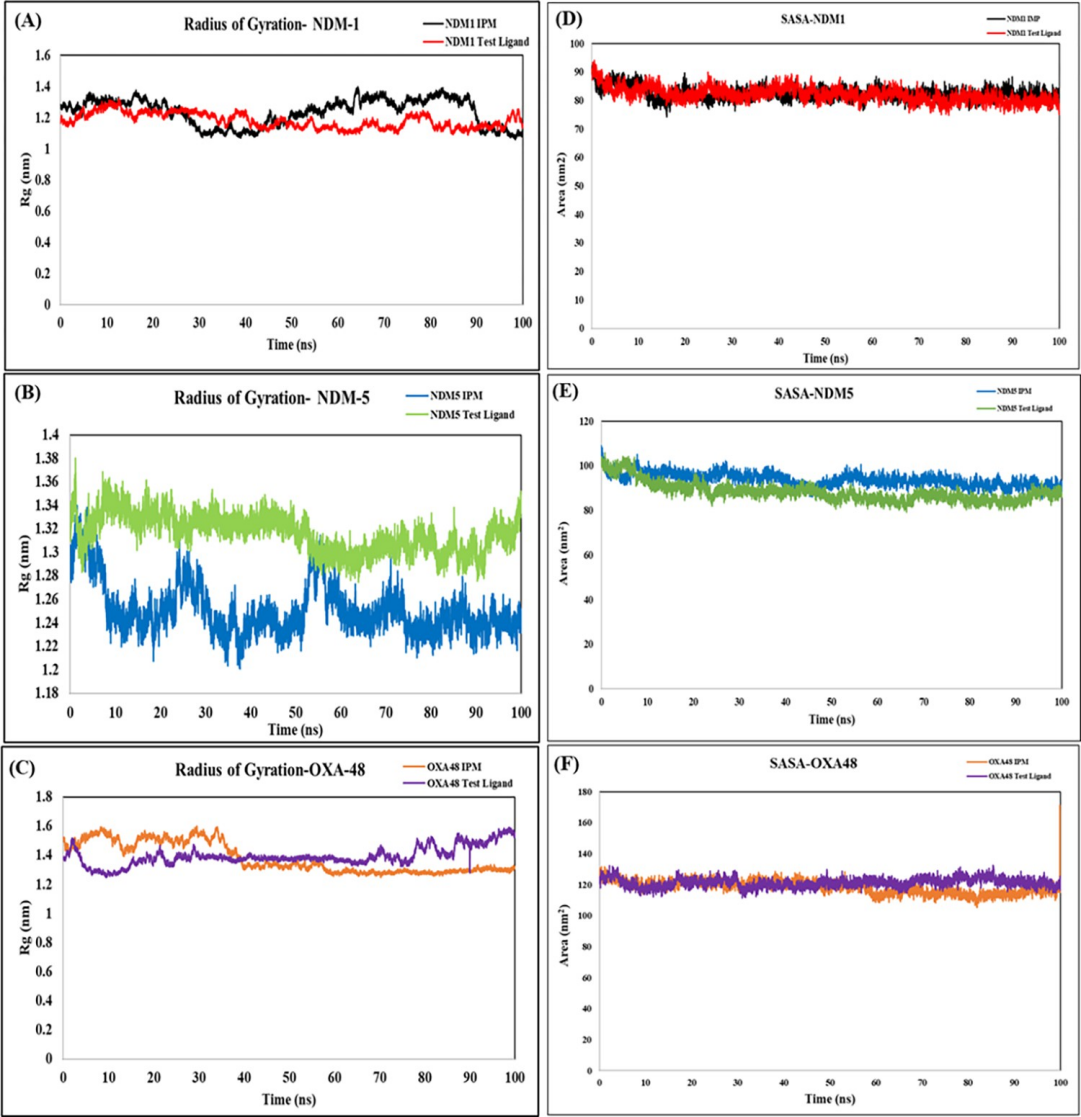
Rg and SASA analysis plots. The Rg graph of the antimicrobial resistance proteins **(A)** NDM-1 **(B)** NDM-5 **(C)** OXA-48 complexes with IPM and test ligand. Time (ns) is displayed on the X-axis and Rg (nm) is displayed on the Y-axis. The solvent accessible surface area (SASA) plot of **(D)** NDM-1 **(E)** NDM-5 and **(F)** OXA-48 protein complexes with IPM and test ligand. Time (ns) is displayed on the X-axis and Area (nm^2^) is displayed on the Y-axis.

Radius of Gyration reveals the complex and protein backbone compactness. The Rg for the NDM-1, NDM-5 and OXA-48 complex with the test ligand was observed to be 1.18 nm, 1.31 nm and 1.39 nm whereas the Rg with the standard drug Imipenem was observed as 1.23 nm, 1.25 nm and 1.38 nm respectively. The pattern of Rg for the protein complexes with both the ligands were similar, indicating a compact configuration of the ligand with the protein (Fig 6A–6C).

Solvent accessible surface area (SASA) is a critical metric tool which determines and quantifies the portion of the protein’s surface that becomes accessible to the surrounding solvent molecules, typically water. SASA analysis provides an insight into hydration properties, binding interactions, stability, and conformational transitions relevant for understanding the protein function. SASA trajectories within the 100 ns simulation observed a mean value of 82.09 nm, 87.67 nm, and 121.30 nm for the proteins NDM-1, NDM-5 and OXA-48 complexes with the test ligand, while the mean SASA trajectories recorded for the proteins with Imipenem were 82.5 nm, 90.9 nm and 118.4 nm respectively. First 40 s observed an elevated SASA value for the protein complex with the test ligand which then was observed to reduce until 100 ns. Reduced SASA overtime suggests stable conformations aiding in stability assessment during MDS. And higher SASA values indicate more hydration because of more solvent exposure thus affecting the stability of the complex (Fig 6D–6F).

**Fig 7:**
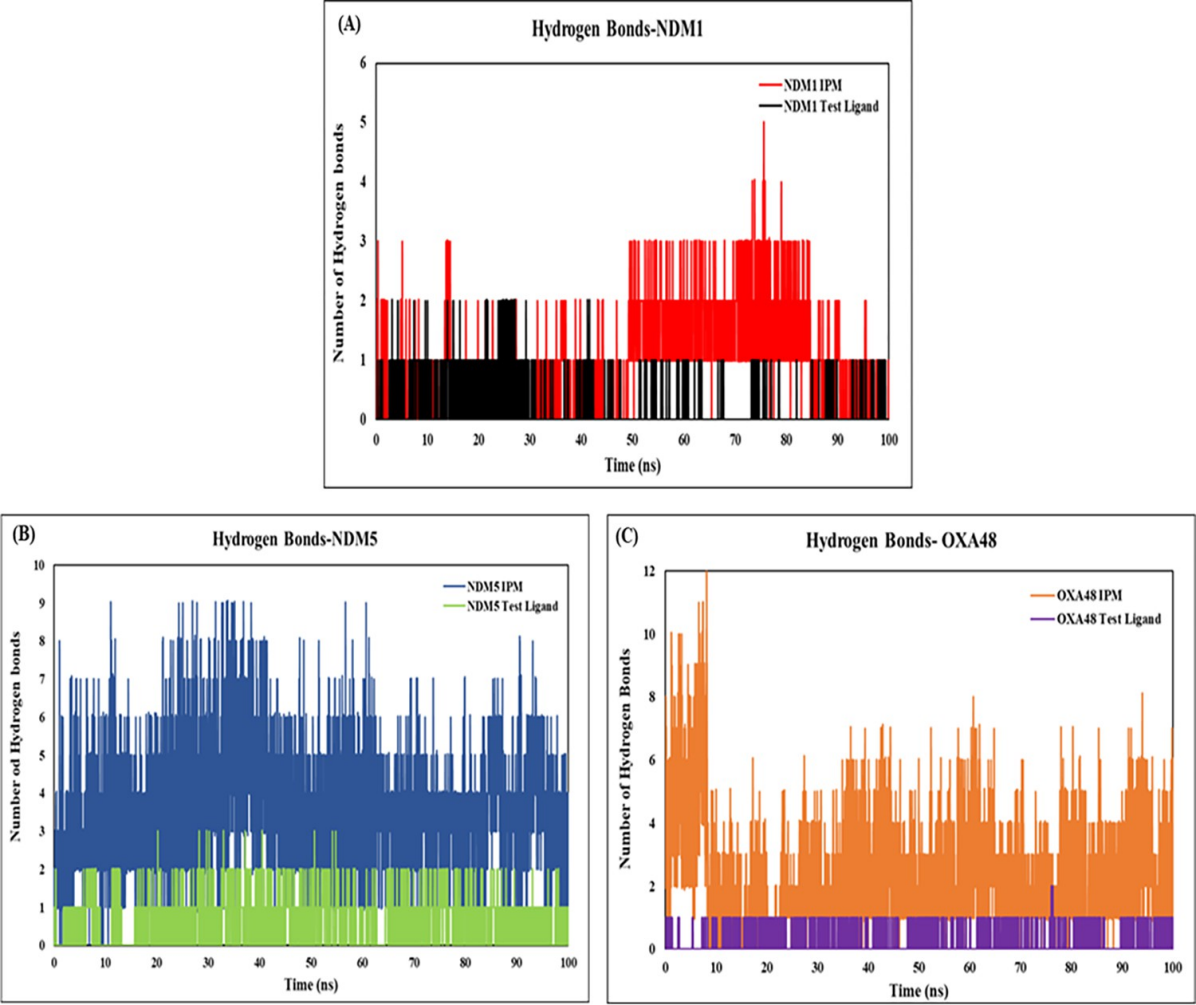
The hydrogen bond interactions of the three protein complexes for 100ns simulation period **(A)** NDM-1 **(B)** NDM-5 and **(C)** OXA-48 complex with IPM and the test ligand.

To evaluate the stability of the protein-ligand complex, the conventional H-bonds were taken into consideration. Overall, the mean H-bonds of the protein complex with the test ligand was recorded as 1.18, 0.75 and 0.24 for the NDM-1, NDM-5 and OXA-48 respectively. Whereas, the average H-bonds of the protein-imipenem complexes were observed to be 0.9, 3.6 and 2.2 respectively for the three proteins. The protein hydrogen bond interactions of Imipenem were observed to be on the higher side when compared to the test ligand (Fig 7A–7C).

**Fig 8:**
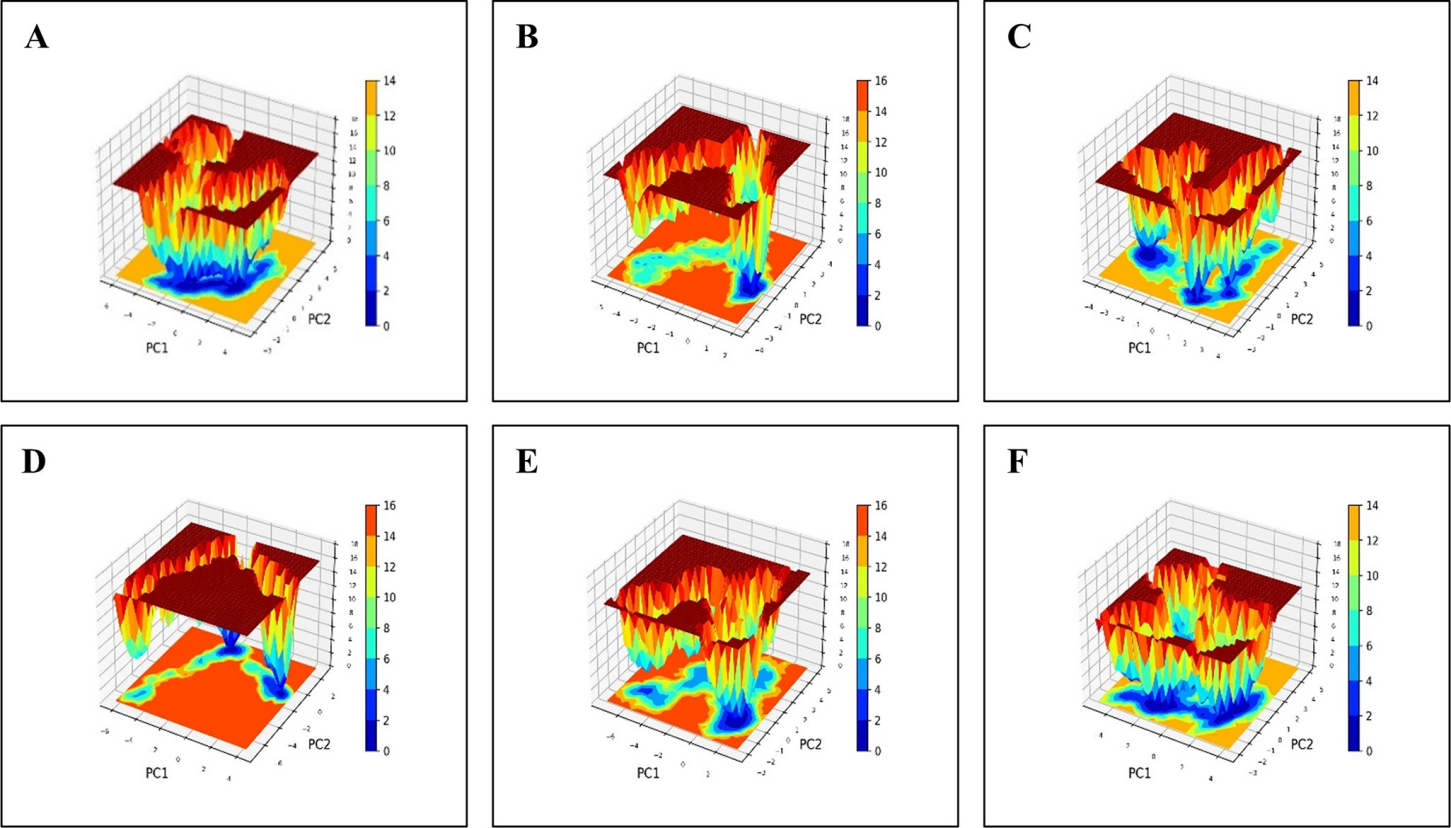
FEL plot. **(A-C)** The free energy landscape analysis of the complexes of AMR proteins NDM-1, NDM-5 and OXA-48 with IPM respectively. **(D-F)** FEL plots of NDM-1, NDM-5 and OXA-48 protein with the test ligand respectively.

**Fig 9:**
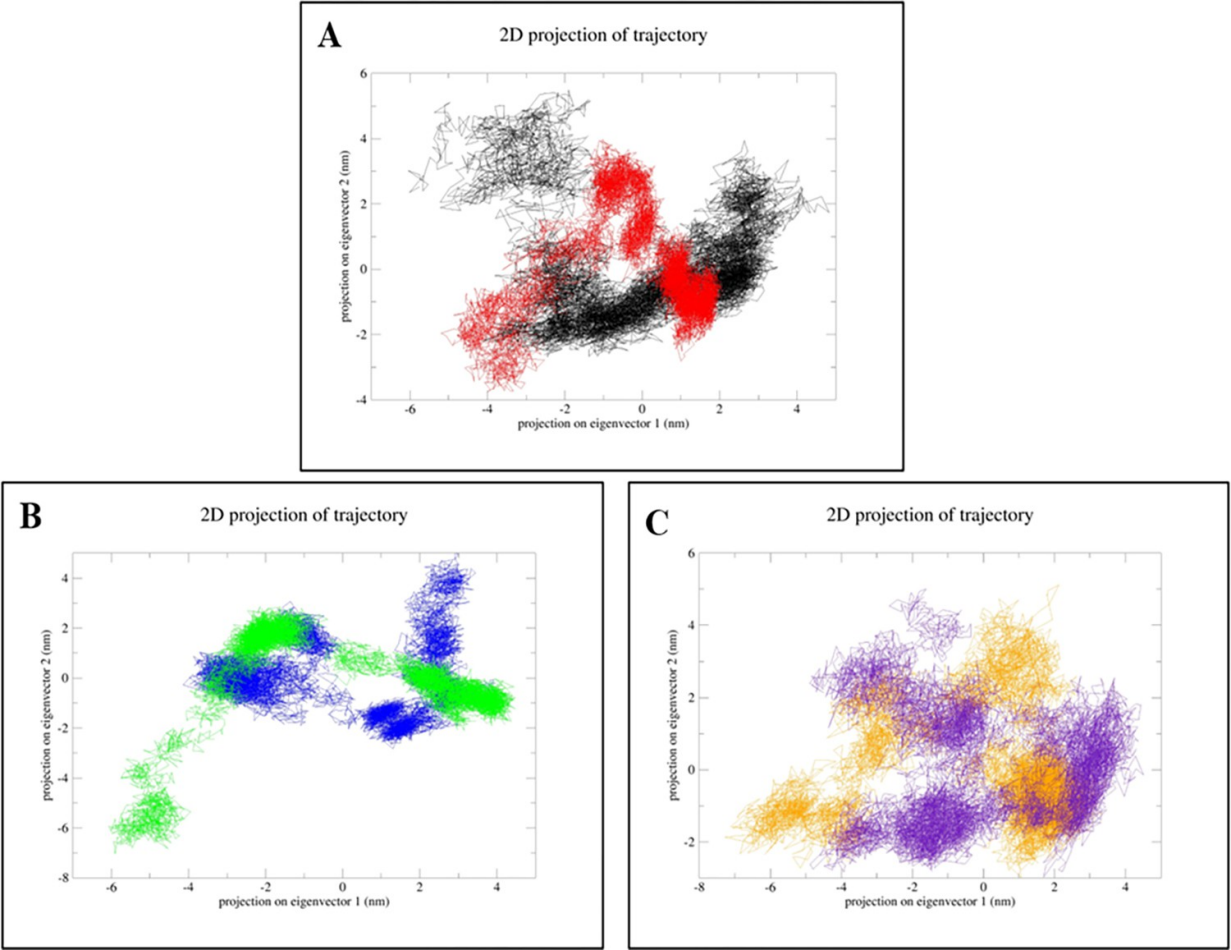
The principal component analysis (PCA) of NDM-1, NDM-5 and OXA-48 protein complexes with Imipenem and test ligand. **(A)** The black color represents the NDM1-IPM complex and red color represents the NDM1-test ligand complex. **(B)** Blue color represents the NDM5-IPM complex and green color represents the NDM5-test ligand complex. **(C)** Orange color represents the OXA48-IPM complex and purple color represents the OXA48-test ligand complex.

The Free energy landscape (FEL) is a three-dimensional plot that follows a color scheme of red, yellow, blue and green to depict the potential energy surface of a system. The FEL plot was constructed to determine the maximum and minimum energy of the three complexes. Each complex has a unique FEL pattern with the red colored spots defining lowest energy and best conformation (Fig 8A–8F). PCA analysis was also applied to determine the structural changes produced by the protein-ligand interactions. To ensure the accuracy of the associated motions, two eigenvectors of each trajectory were compared. To further comprehend the complex conformational shift, a 2D domain was created from the trajectories using the eigenvector values 1 nm for the X-axis and 2 nm at the Y-axis. The PCA figure shows that the eigenvalues of the NDM1-IPM complex range from around -7 nm to 4 nm whereas the NDM1-test ligand ranges from -4 nm to 2 nm. Similarly, the eigenvalues of NDM5-IPM vary from -4 nm to 4 nm, while the NDM5-test ligand ranges from -6 nm to 4 nm. OXA48-IPM variations range from -7 nm to 3 nm, whereas the OXA48-test ligand fluctuates between -4 nm to 4 nm. The PCA plot shows that the test ligand binds to the protein resulting in a more stable-complex structure. Overall, the antimicrobial-resistant proteins of *E*. *coli* form a better stable complex with the test ligand, with a shorter phase space than the protein-IPM complexes (Fig 9A–9C).

The average free binding energy for the protein-IPM and protein-test ligand complexes revealed the lowest energy for all the protein-test ligand complexes when compared to their counterparts with the standard control, Imipenem. Free binding energy determines the measurement of the release of energy during the bond formation between the protein and the ligand, therefore lower the energy better the binding capacity. The van der waals, electrostatic and polar solvation energies are all summed up to form the total free binding energy. The interaction of various energy types, excluding polar solvation energy, facilitated the binding of the compounds to antimicrobial resistance proteins. Among the two compounds studied, the test ligand 235414 exhibited the lowest binding energies: -105.081 ± 14.900 for NDM-1, -90.483 ± 12.335 for NDM-5, and -75.729 ± 10.350 for OXA-48 proteins (Table 6). These results indicate that ligand 235414 may serve as a more effective bactericidal agent against antimicrobial resistance proteins in *E*. *coli* compared to the FDA-approved drug Imipenem. Additionally, the RMSD, RMSF, Rg, SASA, H-Bonds, PCA, FEL, and MM/PBSA analyses confirmed the stability of the protein-ligand complexes throughout the simulation. In summary, Oxacyclohexadecan-2-one shows potential as a superior antimicrobial compound for preventing re-infection and recurrence of urinary tract infections caused by *E*. *coli* due to the emerging antimicrobial resistance.

**Table 6:** The MM-PBSA calculations of binding free energy for the three antimicrobial resistant proteins with Imipenem and test ligand, Oxacyclohexadecan-2-one.

Protein	Ligand	Van der Waals energy (±SD)	Electrostatic energy (±SD)	Polar solvation energy (±SD)	Total binding energy (±SD)
blaNDM-1 (kJ/mol)	Imipenem	-128.768 +/- 10.034	-41.727 +/- 17.847	84.714 +/- 20.120	-98.201 +/- 7.789
	Test ligand	-141.302 +/- 10.063	-6.987 +/- 7.630	58.378 +/- 11.522	-105.081 +/- 14.900
blaNDM-5 (kJ/mol)	Imipenem	-92.897 +/- 15.538	-48.114 +/- 17.771	83.261 +/- 10.302	-70.747 +/- 8.988
	Test ligand	-100.247 +/- 10.497	-9.144 +/- 5.715	50.471 +/- 5.833	-90.483 +/- 12.335
blaOXA-48 (kJ/mol)	Imipenem	-77.442 +/- 15.444	-74.066 +/- 15.680	127.261 +/- 29.155	-35.543 +/- 10.904
	Test ligand	-93.414 +/- 6.025	-6.328 +/- 8.369	36.249 +/- 11.038	-75.729 +/- 10.350

## Discussion

Although globally, the prevalence of carbapenem-resistant *E*. *coli* infections remains relatively low compared to other resistant bacteria, there are concerning trends, especially, in regions like India where the empirical usage of antibiotics are high [30, 31]. Therefore, majority of these resistant *E*. *coli*, often tend to produce enzymes such as NDM, OXA, VIM, IMP, KPC, which break down the beta-lactam ring of the carbapenem drugs, thus rendering the drug inactive [32]. Considering the regional variations of carbapenem resistant *E*. *coli* strains, certain parts of South and East Asia report higher resistance rates, primarily due to the prevalence of NDM-producing *E*. *coli*. Therefore, to decrease the incidence of antimicrobial resistance in UTI, especially in vulnerable groups such as the diabetics, search for more effective and safer therapeutic alternatives are the need of the hour. As the uropathogens that cause UTI in diabetics are multi-drug resistant or pan-drug resistant, scientific screening of naturally derived-compounds as a safer option against the resistance-conferring genes is imperative. Recent breakthroughs, such as in-silico molecular docking and ADME(T) approaches, have proven to be invaluable tools for screening prospective therapeutic drugs in a time and resource effective manner [33].

Our study aimed to find novel therapeutic agents from *M*. *oleifera* seed-derived compounds, given the urgent need for alternatives to current antibiotics like imipenem which is facing reduced efficacy due to resistance [34]. The 3D protein structures of blaNDM-1, blaNDM-5 and blaOXA-48 were successfully modeled and their active site was identified. Among the virtually screened 135 GC-MS derived *M*. *oleifera* compounds, Oxacyclohexadecan-2-one was identified as a promising candidate owing to its favorable binding energies and low toxicity in ADME analysis. The docking scores of Oxacyclohexadecan-2-one was observed to be better than the standard beta-lactam drug, Imipenem, which is used commonly to treat complicated cases of UTI [35]. This is suggestive that Oxacyclohexadecan-2-one could be a potential beta-lactamase inhibitor.

The target proteins NDM-1, NDM-5 and OXA-48 were all monomers with a length of 153 aa, 183 aa and 221 aa respectively. The native 3D protein structures were modeled by SwissModel and obtained as native structures. Out of the total 135 ligands virtually screened by PyRx, the top 10 best ligands chosen exhibited maximum binding energy and zero RMSD depicting no changes in target conformation with the respective proteins. The H-bonds at the binding site were observed to have an interaction with these compounds. Prior to docking the target proteins with a single test ligand, the toxicity of all the top 10 compounds were preferably studied. Therefore, the pharmacokinetics, drug-likeness and the Lipinski rule were checked in the SwissADME server. ProTox online tool evaluated the compounds for their toxicity class by applying filters at each step to identify potential drug-like compounds. One best compound, Oxacyclohexadecan-2-one was finally selected after observing all the findings of the toxicity and ADME analysis.

Imipenem was chosen as the standard control in the study as it is one of the most prescribed Carbapenem class-drugs to treat UTI infections [36]. Imipenem is being widely used empirically in India to treat bacterial infections thereby leading to the development of carbapenamase-producing strains of pathogens [37]. After performing the docking study with the test ligand 235414, the docking scores were compared with that of Imipenem. The test ligand had superior docking scores compared to that of the control. The 2D interactions of all the three protein molecules with Imipenem had relatively a greater number of hydrogen bonds compared to the test ligand. FDA approved drugs such as Imipenem, which is currently being used to treat UTI, have already been extensively modified and tested [38]. While the number of hydrogen bonds influence the stability of the ligand-protein complex, it is not considered as the only factor. Other types of interactions, such as alkyl, pi-pi stacking, and electrostatic interactions, are also essential [39]. Despite Imipenem forming more hydrogen bonds with all the three target proteins, clinically, the resistance to imipenem is well documented, limiting its effectiveness [40]. This is a result of mutation in the target protein that affect the binding site, or the upregulation of efflux pumps that remove the drug from the cell [41]. Apart from the H-bonds, factors such as the RMSD, RMSF and the free binding energy play a crucial role in determining the stability of the ligand-protein complex. With a different interaction profile, the test ligand might not be affected by these resistance mechanisms in the same way. Since the efficacy of Imipenem is disrupted by resistance mechanisms, having a different interaction profile may enable the test ligand to bypass these resistance mechanisms and act as an inhibitor.

Molecular dynamics (MD) simulations are an essential component of computational drug discovery, providing insights that support docking studies [42]. To validate the docking results and investigate the stability of the protein and protein-ligand complexes, MD simulations were performed. The MD simulations for the Imipenem and the test ligand were run for 100 ns. The test ligand-protein complex revealed an overall lower average score compared to the Imipenem-protein complex counterparts, indicating that the RMSD remained stable throughout the simulation. To identify any possible conformational changes of the protein with the ligand during simulation, RMSF was calculated. The overall average RMSF observed for the Imipenem-protein complexes were higher compared to the test ligand-protein complex. A high RMSF value implies that the relevant residue or atom has a lot of flexibility or movement during simulation. This could be because the residue is in a loop region or an unstructured portion of the protein, both of which are more flexible than other sections [43]. A low RMSF value, on the other hand, implies that the residue or atom is very stable and moves little throughout simulation. This is frequently observed in protein core residues or structured regions such as alpha-helices and beta-sheets. Rg analysis to evaluate the overall compactness of the protein-ligand complex revealed a slightly higher overall mean Rg value for the NDM-5-test ligand and OXA-48 test ligand complex. This could be due to various reasons such as the presence of loops or secondary structures in the protein moiety, that confers conformational change during simulation [44]. The presence of water molecules at the binding site could also result in higher Rg values. Since the test ligand, Oxacyclohexadecan-2-one has not been extensively studied before, there is also a possibility that the ligand possesses allosteric effect, affecting the regions beyond active site in turn playing a role on the entire protein-dynamics [45]. Further, to assess the protein’s structural folding and dynamics in an environment with a solvent, SASA analysis was performed. SASA values denoted that the proteins-test ligand complexes had a better solvent-accessible surface area when compared to the protein complex with imipenem. Oxacylcohexadecan-2-one despite forming fewer hydrogen bonds in MDS maintains stable and robust interactions with the target proteins highlighting its potential as an effective antibacterial agent.

PCA analysis was carried out to identify changes in the complex’s conformation due to pressurization and temperature variations [46]. The most important eigenvectors from PCA represent the protein’s precise atomic mobility [47]. The protein-test ligand complexes have a narrower conformational area than the imipenem complex, indicating higher structural stability. FEL analysis revealed that ligand-bound structures improved stability and the density of packing in protein-folding patterns across complexes. PCA and FEL data showed that the test ligand, Oxacyclohexadecan-2-one, formed more stable complexes with beta-lactamase producing proteins than the IPM molecule.

In addition, these findings have been evaluated and validated using MM/PBSA free energy analysis. From the simulated compounds, we noticed that the test ligand had the lowest binding energy with beta-lactamase generating proteins than IPM. In-silico technology can help anticipate the likely active drug, specific ADMET properties, and toxicological implications of medications [48]. The current study used a range of prediction methods for estimating chemical compounds, which could pave the path for the development of novel, safer medicines using an in-silico approach. Our study sets the stage for developing new therapeutic strategies against antibiotic-resistant *E*. *coli* infections, with Oxacyclohexadecan-2-one showing significant promise.

## Conclusion

In this study, the three most prevalent AMR proteins NDM-1, NDM-5 and OXA-48 from uropathogenic *E*. *coli* (UPEC) were computationally screened against 135 phytocompounds derived from *M*. *oleifera* seeds to identify plant-based alternatives to Imipenem for treating complicated UTIs. Among the screened compounds, Oxacyclohexadecan-2-one exhibited superior docking scores, binding energy and, ADME properties compared to Imipenem, highlighting its potential as an effective inhibitor of carbapenemase-producing *E*. *coli*. The presence of additional bioactive compounds, including moringine, pterygospermin, and benzyl isothiocyanates, in *M*. *oleifera* seeds, further supports their potential AMR properties. Quercetin and chlorogenic acid of *M*. *oleifera* also contribute to the plant’s anti-diabetic effects, offering dual therapeutic benefits. Therefore, these findings suggest that *M*. *oleifera* seeds, particularly Oxacyclohexadecan-2-one, could serve as a promising candidate for developing novel treatments for UTIs and rUTIs. Future research, focusing on compound synthesis, in vitro and in vivo evaluations must be done to establish its clinical efficacy and therapeutic applications.

## Supporting information

S1 Data (SDF)

S1 Graphical abstract (TIF)
