# Supplementary figures and images for: Targeting beta-lactamase activity with Oxacyclohexadecan-2-one in carbapenem-resistant uropathogenic E. coli: A molecular simulation approach
Source: PLoS One. 2025 Feb 18;20(2):e0317941. doi: 10.1371/journal.pone.0317941 (PMC11835335; doi:10.1371/journal.pone.0317941)

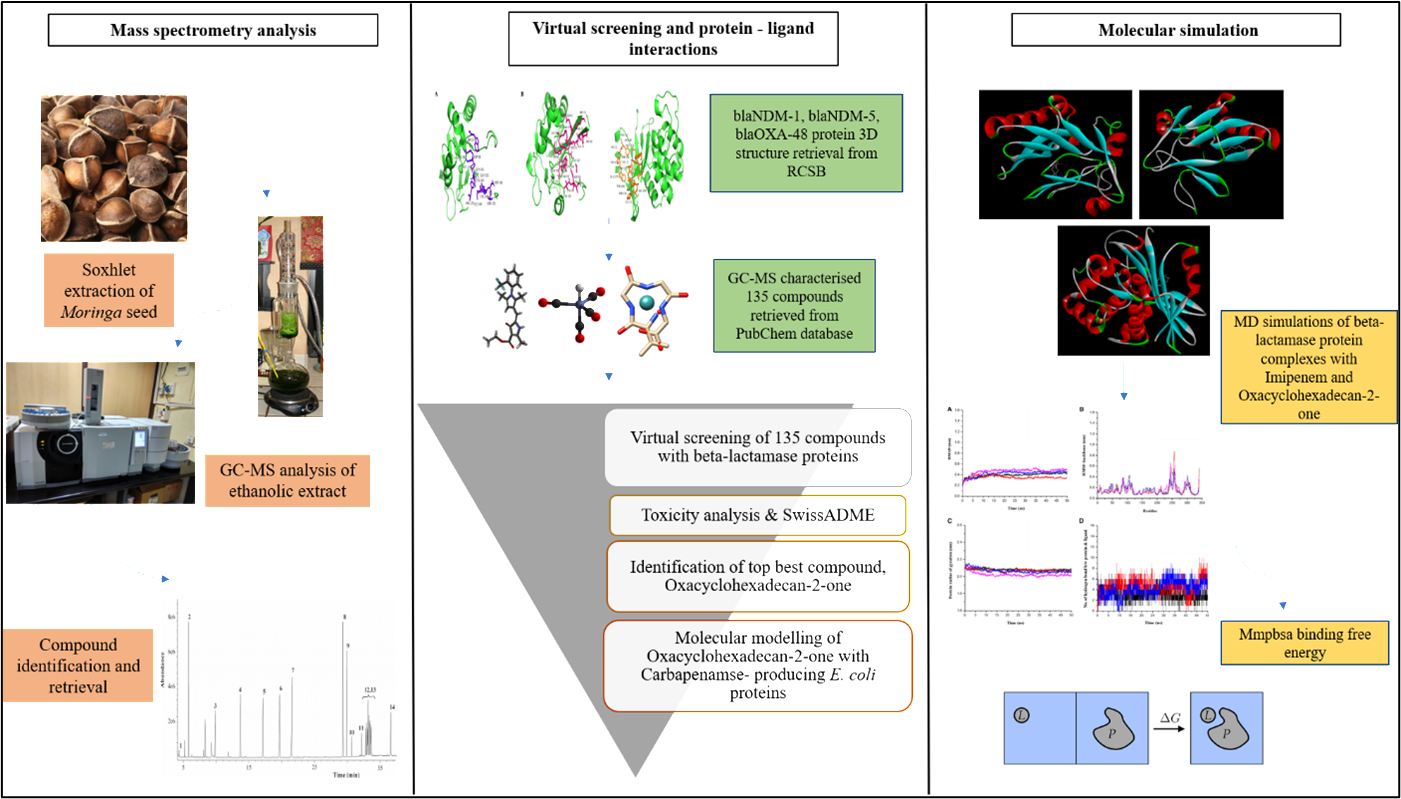

Supplement: S1 Graphical abstract — (TIF) [file pone.0317941.s002.tif]
